# Safety and efficacy of interventional treatment of acute limb ischemia in Germany 2021

**DOI:** 10.1186/s42155-023-00393-8

**Published:** 2023-08-26

**Authors:** Moritz B. Bastian, Jonathan Nadjiri, Joel Wessendorf, Michael Scheschenja, Alexander M. König, Jarmila Jedelska, Andreas H. Mahnken

**Affiliations:** 1 Department of Diagnostic and Interventional, Radiology University Hospital Marburg, Philipps-University of Marburg, Baldingerstrasse 1, 35043 Marburg, DE Germany; 2 Department of Interventional Radiology, Klinikum rechts der Isar, Technische Universität München, Munich, DE Germany

**Keywords:** Lower limb ischemia, Mechanical thrombectomy, Pharmacological thrombolysis, Arterial thrombosis, Embolism

## Abstract

**Purpose:**

Interventional procedures have become a mainstay in the therapy of acute limb ischemia caused by embolism or arterial thrombosis. Treatment options include pharmacological thrombolysis (PT) and mechanical thrombectomy (MT). The aim of this study was to evaluate success and major complication rates of interventional radiological treatments of arterial embolism and thrombosis in Germany in 2021 and to compare their results with accepted international quality standards.

**Materials and methods:**

Data for PT and MT for 2021 was obtained from the quality management system of the German interventional radiological society (DeGIR). 2431 PT and 1582 MT procedures were documented for 2021, with 459 combinations of PT and MT. Data was analysed for technical and clinical success rates, as well as major complication rates such as intracranial bleeding, major bleeding, distal embolization, aneurysm formation, organ-failure and cardiac-decompensation.

**Results:**

PT alone had technical and clinical success rate of 90.21% and 81.08%, respectively. MT alone had technical and clinical success rates of 97.41% and 95.39%, respectively. MT&PT had technical and clinical success rates of 91.07% and 84.75%, respectively. Major complications were: distal embolization (PT:2.02%; MT:1.74%; PT&MT:2.61%), major bleeding (PT:0.94%; MT:1.14%; PT&MT:0.87%), aneurysm formation (PT:0.33%;MT: 1.14%;PT&MT: 0%), intracranial bleeding (PT:0.16%;MT:0%;PT&MT:0.22%), cardiac-decompensation (PT:0.21%;MT: 0.06%;PT&MT:0%) and organ-failure (PT:0%;MT:0.06%;PT&MT:0.22%).

Technical and clinical success rates were higher, while complication rates were lower than the corresponding threshold recommended by the Society of Interventional Radiology for percutaneous management of acute lower-extremity ischemia.

**Conclusion:**

Treatment of arterial embolism and thrombosis performed by interventional radiologists in Germany is effective and safe with outcomes exceeding internationally accepted standards.

## Background

Acute limb ischemia (ALI) is a sudden decrease in blood flow to the limb commonly caused by an embolism or arterial thrombosis. It is a medical emergency that requires immediate treatment to prevent irreversible tissue damage including potential limb loss [1].

Interventional procedures, such as pharmacological thrombolysis (PT) [2, 3] and mechanical thrombectomy (MT) [4], have become a mainstay for the management of ALI. PT involves the use of medications to dissolve the blood clot [3], while MT uses different mechanical devices to physically remove the clot from the artery [5–7]. Both procedures aim to restore blood flow to the affected limb in order to prevent further damage [8]. While these procedures have high success rates, they are also associated with major complications such as intracranial bleeding, major bleeding, distal embolization, aneurysm formation, organ failure, and cardiac decompensation [9, 10]. According to the Society of Interventional Radiology Clinical Practice Guidelines, major complications refer to serious adverse events or outcomes that occur during or after a medical procedure, which can have significant negative impacts on patients' health and well-being [11]. These complications can result in prolonged hospitalization, increased morbidity and mortality, and reduced quality of life. Despite broad utilization of these procedures and thoroughly collected data, there is a lack of in-depth analyses on their efficacy and complication profiles within the context of nationwide German healthcare.

Existing evidence indicates that interventional radiological treatments for acute limb ischemia exhibit high success rates and can improve limb salvage rates [8]. Hospitals performing interventional radiological procedures that are certified by the German interventional radiological society (DeGIR), are expected to report to the quality management system of DeGIR. The quality management system of DeGIR assess pre-, intra-, and post-operative information, i.e., complications and their severity. A previous study analyzing the DeGIR quality management system data showed a nationwide high-quality availability in interventional radiological revascularization procedures in Germany [12]. Furthermore, there is a need to compare success and complication rates reported to DeGIR with international quality standards, which for example have been established by the Society of Interventional Radiology (SIR) in “Quality Improvement Guidelines for Percutaneous Management of Acute Lower-extremity Ischemia” [10].

Thus, the aim of this study is to assess the efficacy and safety of interventional radiological treatments for arterial embolism and thrombosis in Germany during 2021 and to compare these outcomes with established international quality standards. This study's hypothesis posits that interventional radiological treatments for acute limb ischemia in Germany demonstrate high success rates and low major complication rates, which are equivalent to or exceed international quality standards. We aim to provide a status for the safety and efficacy of acute limb ischemia in Germany and to set the foundation for national/international complication and success thresholds.

## Material and methods

### Patient selection

Data of interventional procedures was collected from the quality management system of the German interventional radiology society (DeGIR). All patients included in this study underwent PT and/or MT procedures for acute limb ischemia in Germany during the year 2021. A total of 2431 PT and 1589 MT procedures were documented in 2021. Additionally, 459 PT and MT combined procedures were performed.

### Study design

The study aimed on evaluating the safety and efficacy of interventional treatment of acute limb ischemia in Germany 2021. The basis was the data from the quality management of DeGIR. The data used in this study was collected according to the quality management system standards set by DeGIR, which should ensure accurate and reliable data collection. Data was analyzed for technical and clinical success as well as major complication rates. Procedures were evaluated as clinically successful when alleviation of symptoms was reported after therapy. Alleviation of symptoms were seen as complete or partial pain relief, improvement of muscle function or declined level of paresthesia. Technical success was reported when an angiographically confirmed recanalization of the previously occluded artery could be observed. Major complications were defined as intracranial bleeding, major bleeding, distal embolization, aneurysm formation, organ failure, and cardiac decompensation. Ultimately, the data was compared to accepted international thresholds.

### Statistical analysis

This retrospective study utilized quality management system data to assess efficacy and safety of interventional radiological treatments for acute limb ischemia in Germany. Analyses of the effectiveness and major complication rates was performed using Microsoft Excel (Version 16.34, Microsoft, Redmond, WA, USA). A descriptive statistical analysis was performed. The clinical and technical success rates were hereby analyzed for the efficacy evaluation.

## Results

In 2021 a total of 2431 PT, 1582 MT and 459 MT&PT procedures were reported to the quality management system of DeGIR by 179 different German hospitals (132 for MT, 155 for PT, 79 for PT&MT, respectively). A total of 2431 PT, 1589 MT and 459 MT&PT procedures were included in this report.

### Pharmacological thrombolysis

Acute limb ischemia was treated by PT in 2431 patients. The technical success rate of PT was 90.21% while the clinical success rate was 81.08% (Table 1). The most frequent major complication was distal embolization followed by major bleeding, aneurysm formation, cardiac decompensation and intracranial bleeding with occurrences in 2.02%, 0.94%, 0.33%, 0.21% and 0.16% of procedures, respectively. There was no report of organ failure.

**Table 1:** Displayed are the technical and clinical success rates, and major complications of pharmacological thrombolysis (PT) for acute lower limb ischemia in Germany 2021 in comparison to the thresholds of the Society of Interventional Radiology [10]

**Intervention:**	**Technical Success**		**Clinical Success**		**Major complication**		
PT	Reported [%]	Threshold [%]	Reported [%]	Threshold [%]	Type	Reported Rate [%]	Threshold Rate [%]
Total: 2431	90.21	70	81.08	75	Intracranial bleeiding	0,16	2
					Major bleeding	0.94	10
					Distal embolization	2.02	5
					Organ failure	0	
					Cardiac decompensation	0.21	
					Aneurysm	0.33	

### Mechanical thrombectomy

One thousand five hundred eighty-two patients underwent MT in the therapy of acute limb ischemia. The technical success rate of MT was 97.41% while the clinical success rate 95.39% (Table 2). The most frequent major complication was distal embolization followed by major bleeding, aneurysm formation, organ failure and cardiac decompensation with occurrences in 1.71%, 1.14%, 0.95%, 0.06% and 0.06% of procedures, respectively. There was no report of intracranial bleeding connected to MT.

**Table 2:** Displayed are the technical and clinical success rates, and major complications of mechanical thrombectomy (MT) for acute lower limb ischemia in Germany 2021 in comparison to the thresholds of the Society of Interventional Radiology [10]

**Intervention:**	**Technical Success**		**Clinical Success**		**Major complication**		
MT	Reported [%]	Threshold [%]	Reported [%]	Threshold [%]	Type	Reported Rate [%]	Threshold Rate [%]
Total: 1582	97.41	70	95.39	75	Intracranial bleeiding	0	
					Major bleeding	1.14	
					Distal embolization	1.71	2
					Organ failure	0.06	
					Cardiac decompensation	0.06	
					Aneurysm	0.95	

### Mechanical thrombectomy and pharmacological thrombolysis

Four hundred fifty-nine patients underwent a combined intervention of MT and PT. The technical success rate of MT was 91,07% while the clinical success rate 84.75% (Table 3). The most frequent major complication was distal embolization followed by major bleeding, intracranial bleeding and organ failure with occurrences in 2.61%, 0.87%, 0.22% and 0.22% of procedures, respectively. There was no report of cardiac decompensation or aneurysm formation.

**Table 3:** Displayed are the technical and clinical success rates, and major complications of mechanical thrombectomy (MT) and pharmacological thrombolysis (PT) for acute lower limb ischemia in Germany 2021 in comparison to the thresholds of the Society of Interventional Radiology (SIR) (10)

**Intervention:**	**Technical Success**		**Clinical Success**		**Major complication**		
MT and PT	Reported [%]	Threshold [%]	Reported [%]	Threshold [%]	Type	Reported Rate [%]	Threshold Rate [%]
Total: 459	91,07	70	84,75	75	Intracranial bleeiding	0,22	2^a^
					Major bleeding	0,87	10^a^
					Distal embolization	2,61	2-5^b^
					Organ failure	0,22	
					Cardiac decompensation	0	
					Aneurysm	0	

^a^SIR threshold for MT

^b^SIR Threshold for MT and PT

## Discussion

The findings of this study support the hypothesis that interventional radiology treatments for acute limb ischemia in Germany exhibit high success rates and low major complication rates. Our analysis of quality management system data revealed that pharmacological thrombolysis and mechanical thrombectomy procedures, either performed individually or in combination, were highly effective and safe showing high technical and clinical success rates, as well as low major complication rates. In addition, the results reported to DeGIR indicate that these procedures meet or exceed international quality standards, especially the well-established SIR thresholds [10], providing evidence that the German interventional radiology is delivering high-quality care for patients with acute limb ischemia.

All major complication rates of procedures reported to DeGIR were lower than their respective SIR threshold [10]. Notably not all major complications had a respective SIR threshold such as organ failure, cardiac decompensation and aneurysm for PT, intracranial bleeding, major bleeding, organ failure, cardiac decompensation and aneurysm for MT, and organ failure, cardiac decompensation, and aneurysm for the combination of MT&PT. Even when there was no corresponding threshold, our reported results can be interpreted as safe due to their low occurrence. In MT&PT a complication rate of 2.61% was reported for distal embolization. Having SIR thresholds for distal embolization being 2% for MT and 5% for PT, our results can be seen as effective due to the combination of both procedures. One would expect the complication rate of MT&PT to be align with the sole complication rates of MT and PT, between 2% and 5%.

In the MT group there was no report of intracranial bleeding, which can be explained by the fact that intracranial hemorrhage usually is a typical side effect of blood-thinning medication as seen in the PT and MT&PT groups [13, 14]. The most common major complication was distal embolization, which had the highest incidence in the MT&PT group with 2.61%. A possible explanation for this could be that patients who have the indication for a combined MT and PT procedure usually have a higher level of occlusion and through the combination of mechanical thrombectomy with thrombolysis it could be more likely for a thrombus to drift further distal and potentially lead to distal embolization. Aneurysm formation, organ failure and cardiac decompensation were seen amongst PT and MT. These complications are not the typical procedural complications one would expect. However, as they are reported in the DeGIR database, they should not be withheld as they can occur in the daily interventional routine and can lead to severe consequences.

When comparing our data to other published studies, we found that our results align with previous research demonstrating the efficacy and safety of interventional radiological treatments for acute limb ischemia [15–17]. For example, a systematic review and meta-analysis of endovascular therapy for acute limb ischemia reported a high success rate and low major complication rate, which is consistent with our findings [18]. Comparing our data to the quality standards of the SIR, German hospitals perform PT and MT interventions at a high level exceeding the given SIR-thresholds for the treatment of acute lower-extremity ischemia. However, it is important to note that our study is the first to provide data specific to the German healthcare system, highlighting the importance of analyzing regional/national data to inform clinical practice. Furthermore, the data of the quality management system can be used to establish national/international quality standards.

Despite the strength of our results, there are limitations to our study that must be addressed. Firstly, the retrospective nature of the study limits our ability to control for confounding variables or establish causality. Secondly, the quality management system data used in this study may be subject to reporting biases or inaccuracies, which could affect the validity of our findings. Data provision for the DeGIR quality management system is voluntary and there are regional differences with less input in the city-states [19]. A fair number of missing entries of interventions can be assumed and report of data is influenced by the personal motivation and activity of the participator [20]. Thirdly, the study only includes data from 2021, which may not be representative of long-term trends in interventional radiological treatments for acute limb ischemia in Germany. Lastly, a limitation is the database itself. Besides success rates and major complications, re-intervention- and limb salvage rates are of major importance. Certain points should be added to the quality assessment sheet to preserve high quality of data such as the aspects of re-intervention and limb salvage rates (1- and 6-months follow-up).

## Conclusion

Interventional radiological treatments for acute limb ischemia in Germany are highly effective and associated with low major complication rates exceeding internationally accepted standards. Data of quality management systems can be used to establish high national/international quality standards. Further research is needed to evaluate the long-term efficacy and safety of these procedures to provide German and/or European thresholds for interventional procedures, as well as to identify strategies for optimizing patient outcomes in the context of the German healthcare system. By enhancing our understanding of the efficacy and safety of interventional radiology treatments for acute limb ischemia in Germany, we can ensure that patients receive the best possible care. Furthermore, the results of this study can contribute to the development of evidence-based guidelines for the management of acute limb ischemia, potentially reducing the incidence of major complications associated with interventional radiology treatments.

## Data Availability

The data that support the findings of this study are available from “DeGIR-Deutsche Gesellschaft für Interventionelle Radiolgie” but restrictions apply to the availability of these data, which were used under license for the current study, and so are not publicly available. Data are however available from the authors upon reasonable request and with permission of DeGIR-Deutsche Gesellschaft für Interventionelle Radiolgie.

## Abbreviations

PT: pharmacological thrombolysis
MT: mechanical thrombectomy
DeGIR: German interventional radiological society
ALI: Acute limb ischemia
SIR: Society of Interventional Radiology
